# Myogenous Temporomandibular Disorders: Diagnostic Concepts and Prospective Pilot Study on Extracorporeal Shockwave Therapy

**DOI:** 10.3390/diagnostics13010051

**Published:** 2022-12-24

**Authors:** Dion Tik Shun Li, Kar Yan Li, Yiu Yan Leung

**Affiliations:** 1 Oral and Maxillofacial Surgery, Faculty of Dentistry, The University of Hong Kong, Hong Kong SAR, China; 2 Clinical Research Centre, Faculty of Dentistry, The University of Hong Kong, Hong Kong SAR, China

**Keywords:** temporomandibular disorders, extracorporeal shockwave therapy, maximal mouth opening, parallel group design

## Abstract

The aims of this article are to discuss the current, and potential future directions, in the diagnosis of myogenous temporomandibular disorders (M-TMD), as well as to report a pilot study to investigate the feasibility and clinical outcomes of extracorporeal shockwave therapy (ESWT) in the treatment of M-TMD. Forty-one adult patients presented with M-TMD were recruited into the study and randomized into two groups: Group 1 received ESWT treatment, whereas Group 2 received placebo treatment. The variables investigated were pain, measured by a numerical rating scale (NRS) and mouth opening. Twenty-six patients (Group 1: n = 14, mean age = 45.3 (16.7) years; Group 2: n = 12, mean age = 46.8 (19.7) years) completed 1-year follow up and were included into the final analysis. In both groups, reduction in pain and increase in MO (unassisted maximum, assisted maximum, and pain-free) were seen at post-treatment 1 year. There were more reduction in pain and increase in all MO in Group 1 than Group 2, but statistical significance was not detected. No major complications were encountered in this study. Although significant differences were not seen between groups, this prospective pilot study provided preliminary evidence that ESWT is safe and potentially beneficial in the treatment of M-TMD.

## 1. Introduction

Temporomandibular disorders (TMD) are a group of conditions related to the impaired function of the temporomandibular joints (TMJ) and the associated neuro-muscular system [1]. Common complaints of TMD include clicking in the joint, pain in the TMJs or masticatory muscle and limited mouth opening, which could affect daily functions such as speech and mastication. The origin of the pain and dysfunction could be from the joint, muscles of mastication, or a combination of the two [2]. 

Since TMD of arthrogenous and myogenous nature may have different etiologies, it is important to differentiate between the two in the clinical setting, as the management approach of these conditions may be different [3,4]. Myogenous TMD (M-TMD) is known to mainly affect adult women from age 25–45 years old [5], and may have a higher tendency to seek treatment than those with TMD of arthrogenous origin [6]. Symptoms of M-TMD may include a moderate dull, pressing pain in the masticatory muscle which may become a sharper and more intense pain upon provocation and function [7]. It is known that apart from somatic causes, psychosocial factors may be responsible for the course of development of M-TMD among others [8].

Various treatment options for M-TMD are available, such as jaw exercises [9], medications [10], splint therapy [11], dry-needling [12], botox injection [13], cognitive behavioural therapy [14], and self-care instructions. While clinicians may find favourable outcomes in some of these options, there are those patients that are not fit for a particular treatment. For example, good patient compliance is required for jaw exercises and occlusal splint therapy, and that occlusal splint therapy may not be appropriate in those who also have obstructive sleep apnea (OSA) requiring continuous positive airway pressure (CPAP). In addition, some of the treatment options might produce undesirable effects, such as possible change in facial shape in the case of botox injection. 

Shockwave is a propagating disturbance of great amplitude which travels in a medium and is faster than the speed of sound. First described in the 1980s for the treatment of urolithiasis [15], extracorporeal shockwave therapy (ESWT) has since shown promising results in conditions such as plantar fasciitis [16,17], erectile dysfunction [18], spasticity in post-stroke patients [19], Achilles tendinopathy [19], and chronic calcific tendinitis of the shoulder [20]. Although the exact mechanism of ESWT in its therapeutic applications is unknown, it is speculated that it has an effect on wound healing [21,22]. Recently, ESWT for the treatment of TMD has gained interest. Initial results revealed beneficial outcomes in the treatment of muscle reflex-induced lock jaw with ESWT [23]. ESWT was shown to produce a protective effect on cartilage and subchondral bone structures in the rat model with temporomandibular joint osteoarthritis [24]. In a recent prospective study comparing the effects of ESWT with ultra-short wave (UW) in the treatment of TMD, ESWT resulted in improved response in terms of pain reduction and increased mouth opening in the short-term [25]. However, there are no studies that compare the efficacy and safety of ESWT compared with conventional treatments for M-TMD, and whether it can produce a long-term benefit is unknown at this time.

The aims of this article are to discuss the diagnostic concepts in M-TMD, and to report a prospective pilot study is to assess the feasibility and safety of ESWT in the treatment of M-TMD, and to compare the efficacy of ESWT and placebo therapy in terms of changes in pain score and mandibular function.

Diagnostic Concepts

Currently, the most accepted diagnostic tool for TMD is the Diagnostic Criteria for Temporomandibular Disorders (DC/TMD), which is used for both research and clinical purposes [1]. For M-TMD, diagnosis may include myalgia, local myalgia, myofascial pain, and myofascial pain with referral, which can involve the temporalis, masseter, and other muscles [1] (Table 1). At present, the standard for diagnosis of M-TMD mainly involves clinical examination and history taking, such as palpation of affected muscles and measurement of mandibular function, as opposed to TMD of arthrogenous origin in which imaging may also play a significant role in diagnosis. Magnetic resonance imaging (MRI) is considered the gold standard in the diagnosis of arthrogenous TMD, since disc abnormalities in location and morphology and presence of joint effusion could be readily assessed [26,27,28]. In addition, cone-beam computed tomography (CBCT) may be used to assess any bony pathologies of the mandibular condyle as well as the glenoid fossa [29].

At present, less is known about the imaging approach to the diagnosis of M-TMD. It has been suggested that M-TMD could be caused by injury to the masticatory muscles due to repeated strain from parafunctional habits, resulting in myofascial trigger points [30,31]. A myofascial trigger point has been described as a hyperirritable spot within a taut band of skeletal muscle, which may be painful to palpation and may also result in referred pain [31]. Understanding and locating such myofascial trigger points may be clinically important, as it has been shown that various invasive treatments, such as dry-needling and injection of platelet-rich plasma, may be useful to alleviate symptoms arising from such myofascial trigger points in the masseter muscle [32]. However, the use of imaging modalities is not routinely carried out in the management of M-TMD due to there being insufficient literature to support its application.

Although taut band, which may house myofascial trigger points, is readily palpable by a trained clinician, the detection of those on imaging is often less than straightforward [33]. Although MRI has been suggested to be useful in locating such taut bands in various muscles such as the trapezius [33,34], it is expensive, inconvenient, invasive in the case where contrast agent such as gadolinium is used, and has not been shown to be useful in the muscles of mastication. Another diagnostic imaging modality which has been proposed is ultrasonography (US) which may be more cost-effective, convenient and accessible. Various reports have described the efficacy of US in the identification of myofascial trigger points in muscles such as the lower back and trapezius muscle [35,36,37,38]. However, to the best knowledge of the authors, there are no reports on the identification of taut bands and myofascial trigger points in M-TMD using imaging modalities such as US. A clinical trial on the detection of myofascial trigger points using US in the management of M-TMD is currently underway at the authors centre in an attempt to fill such knowledge gap. The following sections of this paper will focus on a prospective pilot study on ESWT in the management of M-TMD. 

## 2. Materials and Methods

This prospective pilot study was designed according to the CONSORT 2010 statement. Ethical approval was obtained from the Institutional Review Board of the University of Hong Kong/Hospital Authority Hong Kong West Cluster (HKU/HA HKW IRB) (IRB Reference Number: UW 20-704) prior to the start of this study. Written informed consent was obtained from participating subjects. 

### 2.1. Study Design

This was a parallel-grouped clinical trial with balanced randomization (1:1).

### 2.2. Participants

Ethnic Chinese adults presented to the Discipline of Oral and Maxillofacial Surgery, Faculty of Dentistry, the University of Hong Kong for myogenous temporomandibular disorders were considered for recruitment into the current study:

#### 2.2.1. Inclusion Criteria

- At least 16 years of age;
- Pain in the masticatory muscles, headache attributed to TMD, with or without limited mouth opening and pain in the TMJ.

#### 2.2.2. Exclusion Criteria

- Pain in the TMJ only and not involving muscles of mastication;
- Active infection in the TMJ region;
- Systemic rheumatic diseases;
- Significant systemic diseases, such as uncontrolled hypertension, history of stroke within 6 months, and unstable angina;
- Craniofacial syndromes;
- Previous operations in the TMJ.

After an initial clinical examination and confirmation of the diagnosis of M-TMD (DTSL), the patients were prescribed a 2-week course of non-steroidal anti-inflammatory drugs (NSAIDs) (Ibuprofen 400 mg TDS), or paracetamol (500 mg QID) if NSAIDs were contraindicated. If the clinical symptoms were refractory to medication (no reduction in pain score), the patients were then be recruited into the study. 

### 2.3. Pre-Treatment Assessment

#### 2.3.1. Clinical Diagnosis

The clinical diagnosis was based on the Diagnostic Criteria for Temporomandibular Disorders (DC/TMD) [1]. Any type of pain in the masticatory muscle (myalgia, tendonitis, myositis, spasm) and any headache attributed to TMD were recorded. 

#### 2.3.2. Assessment of Pain

Pre-treatment pain symptoms, both at rest and during mandibular movement, was measured with a 11-point (0–10) numerical rating scale (NRS) adopted from the Graded Chronic Pain Scale [39], with 0 indicating no pain, while 10 indicates maximum pain. 

#### 2.3.3. Assessment of TMJ Function

Mouth opening (MO, pain-free, unassisted maximum, and assisted maximum, measured with a ruler between the incisal edges of the upper and lower incisors, minus the overbite), was measured in millimetres in the same way using the midpoints of the upper and lower incisors as references. 

### 2.4. Interventions

The two arms of intervention are ESWT versus placebo. After palpation and identification of the region of pain, the patients were blinded with regards to which group they had been allocated to, and were treated in the following manner:

#### 2.4.1. Group 1: Extracorporeal Shockwave Therapy

In the ESWT group, Focused ESWT (DUOLITH^®^ SD1 T-TOP, Storz Medical) was applied at 0.15 mJ/mm^2^ and stand-off II as per manufacturer’s recommendations for craniomandibular dysfunction (CMD) to the painful side of the masseter muscle by a single operator (DTSL), for three sessions delivered at one-week intervals. At each session, 500 pulses were delivered to the masseter muscle. 

#### 2.4.2. Group 2: Placebo

In the placebo group, the handpiece of the ESWT was connected to a placebo stand-off with zero energy output so that no shockwave was transmitted to the patient. The procedure was carried out in the same way as in the ESWT. The patients were treated once a week for 3 weeks.

### 2.5. Outcomes

The primary outcome measure was pain symptoms in 6 weeks, as measured with a numerical rating scale (NRS). The secondary outcome measure was TMJ function.

#### 2.5.1. Assessment of Pain

Post-treatment assessment of pain symptoms was measured with an NRS 1 week after each treatment session and at 6 weeks, 3 months, 6 months and 1 year after the first treatment session. 

#### 2.5.2. Assessment of Mouth Opening

Post-treatment assessment of pain-free mouth opening was carried out 1 week after each treatment session and at 6 weeks, 3 months, 6 months and 1 year after the first treatment session. Post-treatment assessment of maximum unassisted and maximum assisted mouth opening was performed at post-treatment 1 year.

#### 2.5.3. Complications

All intra-operative, immediate, early post-operative, and late post-operative complications were recorded. 

### 2.6. Randomization

Recruited patients were randomized into one of the two study groups by a simple randomization procedure. Using a computer program, a randomization table was generated. The allocation sequence was kept concealed in sequentially numbered, opaque and sealed envelopes. Upon obtaining the study consent from the participants, the surgeon in-charge would open the sealed envelope containing the allocation sequence.

### 2.7. Statistical Methods

Statistical analysis was performed using SPSS 28 software (IBM Corp., New York, NY, USA). For continuous variables, Shapiro-Wilk normality test was performed to test if the data followed normal distribution. For testing the differences at the same time interval between groups, independent *t*-test (or Mann-Whitney test if normality of the data was not fulfilled) was used. Comparison between baseline and other follow-up time points were performed with the paired-sample *t*-test (or Wilcoxon Signed Ranks test if normality of the data was not fulfilled). For the analysis of multiple comparisons at other follow-up time points and multiple comparisons between baseline and other follow-up time points, Bonferroni correction was used. *p* < 0.05 was considered statistically significant.

## 3. Results

**Figure 1:**
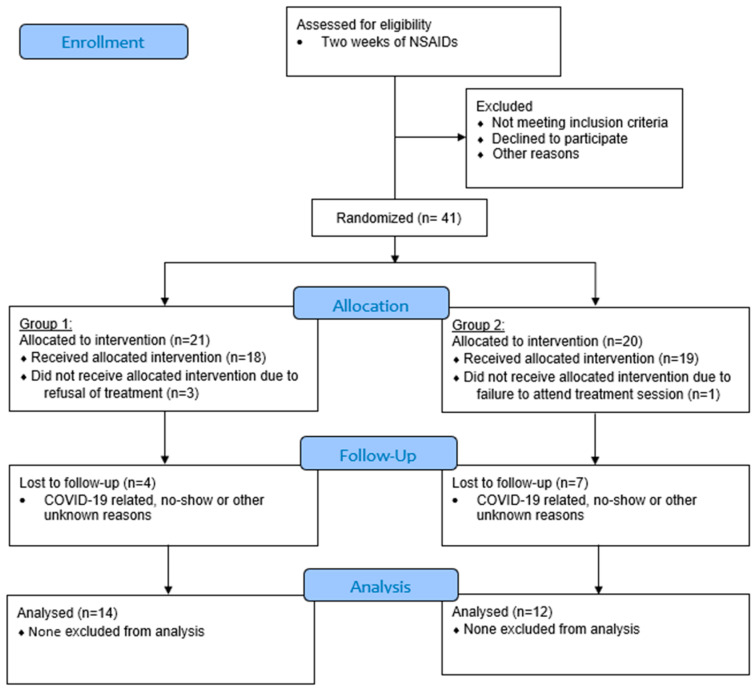
CONSORT flow diagram (version 2010) of subject enrolment, allocation, and follow-up. Group 1: extracorporeal shockwave therapy (ESWT); Group 2: placebo.

Patients whose symptoms of M-TMD were not alleviated by NSAIDs were recruited into the study. A rolling recruitment and randomization strategy were employed in anticipation of a sizable dropout due to the COVID-19 pandemic. A total of 41 patients were initially recruited into the study. Of these, 21 patients were allocated to Group 1 (ESWT) and 20 patients were allocated to Group 2 (placebo). Loss of follow-up occurred in various time-points. The final number of patients who completed the 1-year follow-up schedule included 26 patients: 14 patients in Group 1 and 12 patients in Group 2 (Figure 1).

### 3.1. Patient Characteristics

Demographic characteristics of the final study sample is shown in Table 2. Male patients consisted of 14.3% in Group 1 (n = 2), and 25% in Group 2 (n = 3). The mean age at the time of recruitment was 45.3 (16.7) and 46.8 (19.7) years, respectively, for Group 1 and 2. The mean duration of pain in months at recruitment was 33.5 (36.2) and 42.6 (43.1) for Group 1 and 2, respectively. Other variables are presented in Table 2. There was no significance in any of the demographic characteristics between the two groups.

### 3.2. Pain

**Figure 2:**
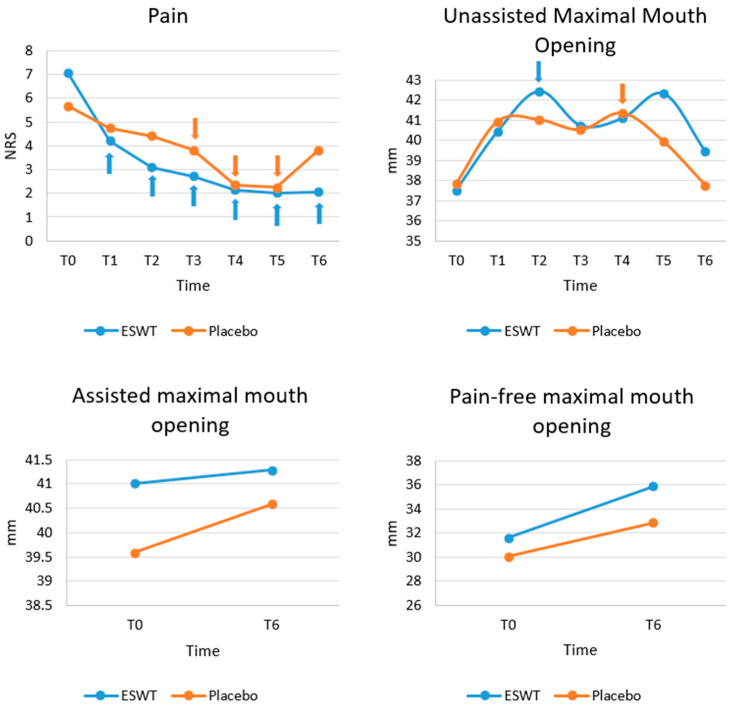
Progression of clinical variables (pain, unassisted maximum mouth opening, assisted maximum mouth opening, pain-free mouth opening) at different time points. Although greater reduction in pain and increase in mouth opening was seen in Group 1, there was no statistically significant difference of any variables at any time point between the two groups. For within group analyses, those values with significant difference compared to baseline (T0) are marked with arrows with corresponding colours. NRS, numerical rating scale; MO, mouth opening; T0, baseline; T1, 1 week after the first session; T2, 2 weeks after the first session; T3, 3 weeks after the first session; T4, 6 weeks after the first session; T5, post-treatment 3 months; T6, post-treatment 1 year.

Figure 2 shows the progression of clinical outcomes (pain, unassisted maximal mouth opening, assisted maximal mouth opening, and pain-free mouth opening), based on the raw data. A normality test showed that pain did not follow a normal distribution (*p* < 0.05) and thus non-parametric tests were performed. 

At baseline (T0), there was no significant difference in pain between Group 1 and Group 2 (*p* = 0.063). There was a greater reduction in pain in the ESWT group than the placebo group at subsequent time points. However, there was no significant difference between the two groups in terms of pain at any follow up time points after Bonferroni correction. Within each group, pain dropped over time significantly compared to baseline. Within Group 1, after Bonferroni correction, significant differences were observed between T0 and T1 (*p* = 0.024), between T0 and T2 (*p* = 0.013), between T0 and T3 (*p* = 0.020), between T0 and T4 (*p* = 0.002), between T0 and T5 (*p* = 0.020) and between T0 and T6 (*p* = 0.013). However, within Group 2, after Bonferroni correction, significant differences were only detected between T0 and T3 (*p* = 0.042), between T0 and T4 (*p* = 0.019), and between T0 and T5 (*p* = 0.0030) (Figure 2). 

### 3.3. Mouth Opening

A normality test showed that unassisted MO did not follow a normal distribution (*p* < 0.05) and thus non-parametric tests were performed. At baseline (T0), there were no significant differences in unassisted maximum MO (*p* = 0.816), assisted maximum MO (*p* = 0.659) and pain-free MO (*p* = 0.725) between the two groups. Then, at all follow-up time points, after Bonferroni correction, no significant differences were detected in unassisted maximum MO, assisted maximum MO, and pain-free MO between the two groups. Within each group for unassisted maximum MO, the only significant difference was found between T0 and T2 in Group 1 (*p* = 0.029) and between T0 and T4 in group 2 (*p* = 0.039) after Bonferroni correction. No significance time changes in assisted maximal MO and pain-free were found within both groups (Figure 2). 

### 3.4. Complications

One patient in Group 1 (male, age 40 years) complained of increased pain which radiated to the temporalis and neck one day after the application of ESWT. He was given paracetamol and the pain subsided the next day. There were no other complications observed.

## 4. Discussion

The diagnosis and management of M-TMD are not always straightforward due to the little understanding of the value of other investigations apart from clinical examination. At present, there is an obvious gap in the literature concerning the use of imaging in the diagnosis of M-TMD. Moreover, there is no consensus on which treatment options are superior to others, as no single treatment modality has consistently provided predictable outcomes [40]. Thus, the management for any individual patient may often be based on a trial-and-error approach and may comprise of a combination of treatment options. Apart from the potential clinical efficacy that a particular treatment modality may be able to deliver, other important considerations in selecting treatment options include safety, convenience, cost-effectiveness, patient compliance, and the possibility to be combined with other treatment modalities.

The result of this prospective pilot study has shown that ESWT is safe and is a potentially beneficial treatment option in the management of myogenous TMD. Reduction in pain and improvement of mouth opening were found to be superior in the ESWT group compared to the placebo group, though these findings were not statistically significant and was likely attributed to the small sample size in this pilot study. 

Currently, there are various treatment options for M-TMD that are commonly employed by clinicians and may be considered conventional options. These include occlusal splints [41,42], physical therapy [11], counselling therapy [43,44], and botulinum toxin injection [12,13,45]. Other treatments that have been mentioned in the literature but may be less common include dry-needling [12,40], low level laser [46], and acupuncture [12,47]. For pain relief of myofascial trigger points in the trapezius muscle, dry-needling and low-level laser have shown promising results [48]; however, whether this can be applied to myofascial trigger points in M-TMD is unknown. In a recent systematic review and network meta-analysis of randomized clinical trials, it was found that manual therapy, counselling therapy, occlusal splint, and botulinum toxin injection may have a positive effect in the management of M-TMD in both the short and intermediate term [40]. On the other hand, another systematic review has found that placebo effect may be responsible for the positive outcomes seen in some other treatment options, such as acupuncture and dry needling [49]. However, the evidence of these findings is mostly of low quality due to the limitations of the studies included.

ESWT may be an emerging novel treatment modality in the management of M-TMD and may be offered in conjunction of other available treatments. ESWT may have a positive effect on wound healing [21,22] which may be beneficial as an additional treatment option for M-TMD. Although ESWT has been describe as a therapeutic option for multiple applications [16,16,17,18,19,20], to the authors’ best knowledge, there are no other studies in the literature that compare ESWT with other conventional treatment options of M-TMD, nor any other studies that describe its safety and feasibility. A comparative study with a 4-week follow up has shown that ESWT may be beneficial in the management of temporomandibular joint disorder, when compared to ultrashort wave (UW) applied to the TMJ, in terms of reduction in pain and improvement of mandibular function [25]. The results of this study suggest that positive outcomes may also be applicable in the treatment of M-TMD and when compared to conventional treatments of M-TMD. However, future prospective studies with a larger sample size may be able to detect any statistical significance.

The current prospective pilot study provides preliminary information regarding ESWT in the treatment of M-TMD; however, there are several limitations. First of all, the sizable dropout rate may represent potential bias in this study, as patients with different traits or resultant clinical outcomes may be more prone to dropout. Moreover, as M-TMD may represent a chronic pain syndrome with patients consulting multiple clinicians and have attempted various treatment options, many patients recruited in this study had received prior treatments for M-TMD. In addition, as a pilot study, the post-operative accessor (DTSL) was the same as the treating clinician and thus was not blinded. Another notable finding was that significant clinical improvement was also seen in the placebo group which suggests that, similarly to other treatment modalities for M-TMD as mentioned above, the placebo effect likely played a role in this study.

## 5. Conclusions

In conclusion, provided the safety, convenience, and likely potential clinical benefit of ESWT in the treatment of M-TMD suggested by the results of this prospective pilot study, it is worthwhile to explore this application further with well-designed future prospective trials with larger sample sizes.

## Figures and Tables

**Table 1:** Classification of myogenous temporomandibular disorders (M-TMD) according to the Diagnostic Criteria for Temporomandibular Disorders (DC/TMD).

Classification of M-TMD	Clinical Findings
Myalgia	Familiar pain in the masseter and temporalis upon palpation or mouth opening
Local myalgia	Familiar pain in the masseter and temporalis localized to the site of palpation
Myofascial pain	Pain in the masseter and temporalis spreading beyond the site of palpation but within the confines of the muscle being palpated
Myofascial pain with referral	Pain in the masseter and temporalis beyond the confines of the muscle being palpated

**Table 2:** Demographic characteristics of the final analysed sample.

	Group 1: ESWT	Group 2: Placebo
Sample size, n	14	12
Male, n (%)	2 (14.3)	3 (25)
Female, n (%)	12 (85.7)	9 (75)
Mean age in years (SD)	45.29 (16.7)	46.75 (19.7)
Duration of pain in months (SD)	33.5 (36.2)	42.58 (43.1)
Pain (NRS) (SD)	7.07 (1.7)	5.67 (1.5)
MO (pain free, mm) (SD)	31.57 (11.9)	30.08 (7.9)
MO (max unassisted, mm) (SD)	37.5 (8.1)	37.83 (7.4)
MO (max assisted, mm) (SD)	41 (7.9)	39.58 (7.5)
Painful conditions (%)		
Arthralgia	5 (35.7)	7 (58.3)
Myalgia	10 (71.4)	10 (83.3)
Myofascial pain with referral	4 (28.6)	2 (16.7)
Headache attributed to TMD	6 (42.9)	3 (25)
Non-painful conditions (%)		
DDWR	6 (42.9)	5 (41.7)
DDWR with intermittent locking	1 (7.1)	3 (25)
DDWOR with limited mouth opening	0	1 (8.3)

ESWT, extracorporeal shockwave therapy; SD, standard deviation; NRS, numerical rating scale; MO, mouth opening; mm, millimetres; TMD, temporomandibular disorders; DDWR, disc displacement with reduction; DDWOR, disc displacement without reduction.

## Data Availability

Data is available in the manuscript.
